# First insights into the physiological status and gut microbiota of free-grazing Tibetan goats in Kangding, Western China

**DOI:** 10.5455/javar.2026.m1036

**Published:** 2026-03-30

**Authors:** Jian Huang, Haiyue Xiao, Xi Liu, Wenwu Gao, Yao Pan, Yuxia Wang, Li Ma, Shengping Long, Yan Li, Jiuhong Heng

**Affiliations:** 1 College of Animal Husbandry and Veterinary Medicine, Southwest Minzu University, Chengdu 610041, China; 2 Ganzi Institute of Animal Husbandry, Ganzi Tibetan Autonomous Prefecture Yak Industry Development Center, Kangding, Sichuan 626000, China; 3 Animal Disease Prevention and Control Center, Bureau of Agriculture, Animal Husbandry, Rural Affairs and Science & Technology, Xinlong County, Sichuan 626800, China

**Keywords:** Extensive goat husbandry, physiological health status, gastrointestinal microbiota profiling, seasonal variation

## Abstract

**Objectives:** This study assessed the health status, morphometric traits, and gastrointestinal microbiota of Tibetan goats reared under traditional free-range conditions in Kangding, western China, without antibiotics, anthelmintics, or vaccinations.

**Materials and Methods:** Five adult Tibetan goats were assessed for body weight, linear measurements, and blood parameters. Microbial communities from six gut segments of the gastrointestinal tract were characterized using *16S rRNA* gene sequencing. To examine seasonal dynamics, rectal fecal microbiota from eight additional goats, sampled during winter and summer, were compared.

**Results:** Despite fluctuating forage availability, growth traits were comparable to those reported for established meat goat breeds. Most hematological and serum biochemical values fell within normal physiological ranges, though mild deficiencies in certain minerals were observed, accompanied by geophagic behavior. The gut microbiota exhibited clear spatial structuring along the digestive tract, with Firmicutes and Bacteroidota as the dominant phyla and higher microbial richness in the hindgut. Seasonal differences were evident in the rectal fecal microbiota, with higher relative abundance of *Escherichia–Shigella* in winter and increased levels of Prevotella and the *Christensenellaceae_R-7_group* during summer. Functional predictions suggested greater nutrient transport and signaling capacity in the small intestine, whereas the hindgut showed stronger metabolic activity, particularly in fermentation-related pathways.

**Conclusions:** Tibetan goats demonstrate notable physiological and microbial adaptability under extensive management. However, seasonal nutritional constraints may pose subclinical health risks. Integrating routine monitoring of physiological and microbial indicators could help safeguard animal welfare, maintain meat quality, and support the long-term sustainability of traditional pastoral systems in this region.

## 1. Introduction

Natural goat grazing plays a pivotal role in sustaining pastoral livelihoods in mountainous regions, embodying a model of reasonable local resource use aligned with the circular economy model [1]. In Kangding District of Ganzi Tibetan Autonomous Prefecture—situated on the southeastern edge of the Qinghai-Tibet Plateau—the rugged topography has long shaped traditional livestock practices. Within this landscape, Guza Town, located along the Dadu River at an elevation of approximately 1,500 m, features a distinct dry-hot valley microclimate characterized by mild temperatures (8–22°C), high relative humidity (60–90%), and exceptional plant biodiversity [2]. These favorable environmental conditions support year-round natural grazing, which remains the cornerstone of the local meat-goat production system.

To preserve traditional husbandry practices and safeguard the distinctive flavor profile of goat meat, many local herders deliberately eschew intensive practices such as backyard fattening, routine vaccination, and the prophylactic use of antibiotics or anthelmintics, adhering instead to principles of ecological farming. Under such systems. Under this system, indigenous Tibetan goats are typically raised for up to four years and slaughtered around the Winter Solstice (December 21st), based on a longstanding cultural belief that extended aging enhances meat quality. However, this prolonged free-range rearing—conducted without preventive health intervention—exposes animals to chronic health challenges, including nutrition imbalances, endemic parasitic infections, and sustained physiological stressors [3]. Collectively, these factors may undermine metabolic homeostasis, immune competence, and overall body condition.

While semi-intensive systems that alternate between confinement and pasture are increasingly adopted in more economically developed areas, full-time natural grazing remains dominant in remote mountainous communities where access to veterinary services, feed supplements, and infrastructure is limited. At the same time, growing concerns are emerging regarding the ecological footprint of extensive grazing. In already fragile ecosystems experiencing vegetation loss and environmental degradation, unregulated grazing pressure may accelerate grassland deterioration and compromise ecosystem resilience [4, 5].

Against this backdrop, a comprehensive assessment of the health status and gastrointestinal microbiota of Tibetan goats managed under traditional grazing regimes is urgently needed [6]. We hypothesized that nutritional status and environmental factors jointly shape the physiological health and gut microbiota composition of this goat breed and that these traits are closely linked. To address this, we integrated body morphometry, hematological and biochemical analyses, and gut microbiota profiling to establish a comprehensive baseline for assessing animal well-being and guiding management practices [7, 8, 9, 10]. To date, no study has concurrently evaluated the physiology and gut microbiota of free-grazing Tibetan goats in such resource-constrained environments. Therefore, we aim to bridge this gap by generating integrated physiological and microbial data and providing a scientific foundation for health interventions that align with traditional pastoral practices and support sustainable livestock production in highland agroecosystems.

## 2. Materials and Methods

### 2.1. Ethical approval

The protocol was approved by the experimental animal management committee of the Southwest Minzu University (2023MDLS041).

### 2.2. Animals and sampling

**Supplementary Figure 1.**
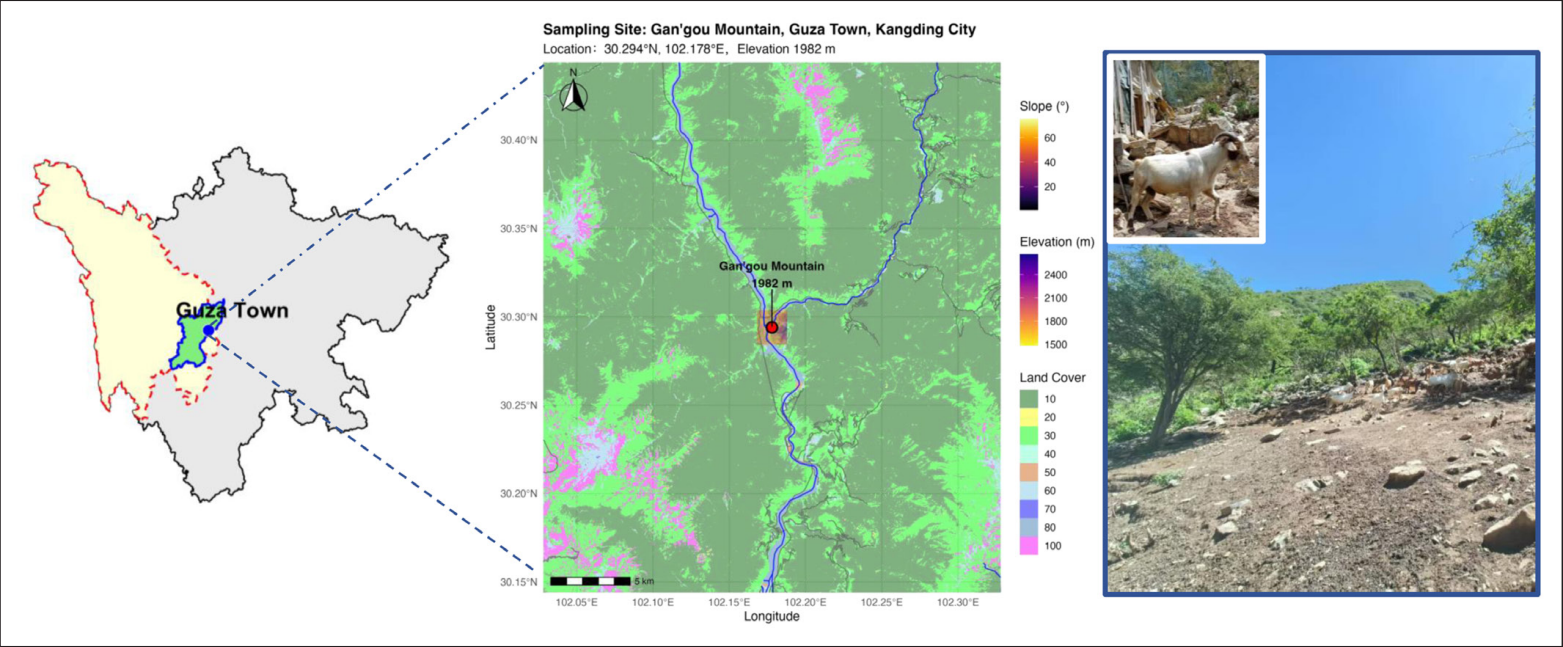
Geographic location and topography of the sampling site for cross-bred Tibetan goats. The sampling site was located in a mountainous river valley near the Dadu River at an elevation of 1,982 meters (Gangou Mountain), where cross-bred Tibetan goats were raised under free-grazing conditions. The green-shaded area on the map denotes Kangding City, the yellow-shaded area corresponds to Ganzi Tibetan Autonomous Prefecture, and the grey area outlines the boundaries of Sichuan Province.

Five clinically healthy, cross-bred Tibetan goats (castrated males, 3.5 years old) grazing on Gangou Mountain (1,982 m elevation) by the Dadu River in Guza Town, Kangding City, Ganzi Prefecture, Sichuan Province, were enrolled, and they were selected from a single herd based on accessibility during the local slaughter season (Supplementary Figure 1). Blood and gut content samples were collected aseptically at slaughter. Their body weight and linear measurements were taken before morning grazing following established protocols (e.g., body condition scoring) [11]. Additionally, paired rectal fecal samples were collected from the same eight goats (3 years old, four castrated males, and four females) during both winter (January) and summer (July). All gut content and fecal samples were immediately kept on dry ice for transport and then stored at –80°C until DNA extraction.

**Figure 1.**
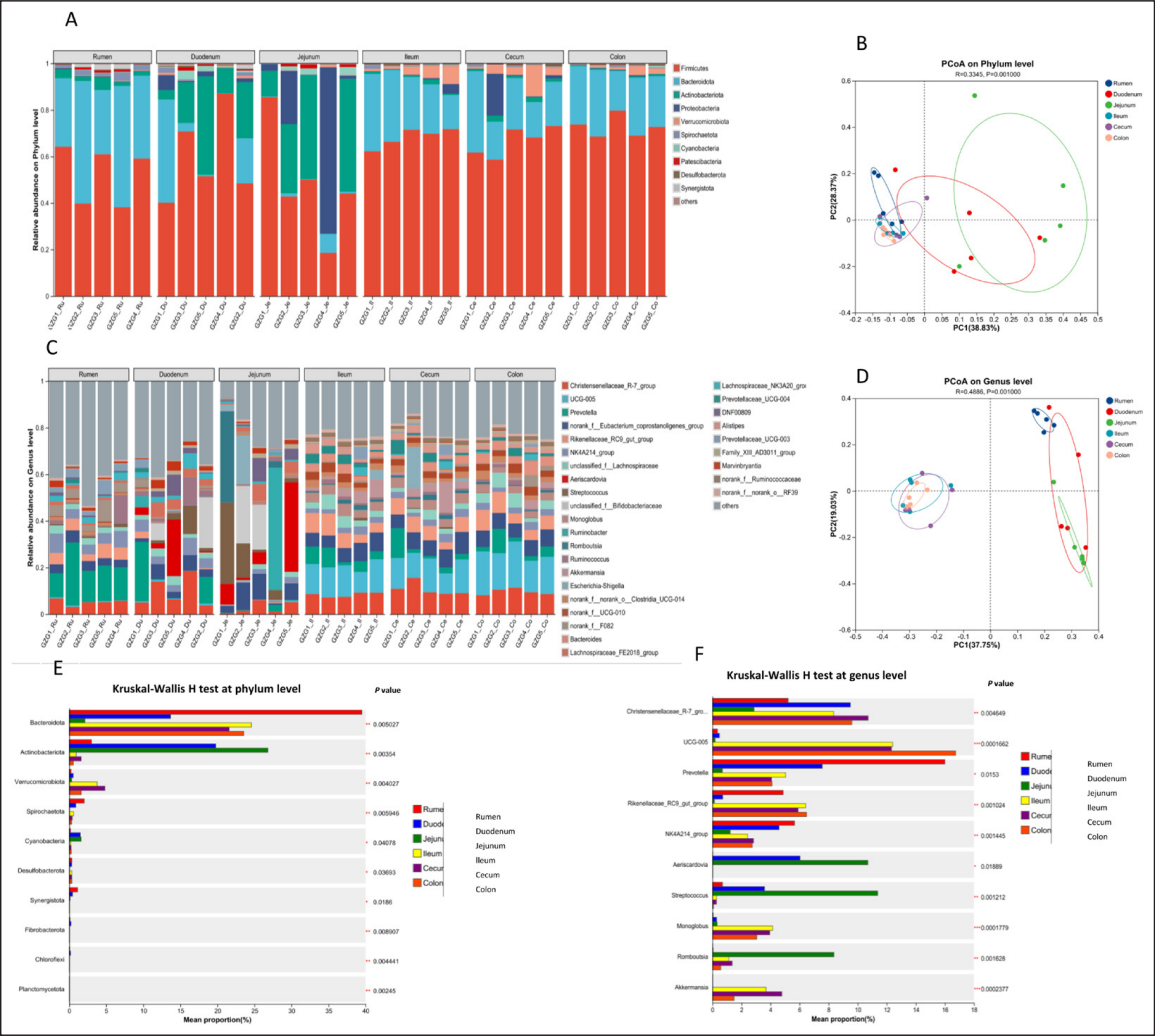
**Beta diversity and taxonomic composition of the gastrointestinal microbiota across six gut segments in Tibetan goats (*n* = 5)**. Stacked bar charts show microbial community composition at the phylum level (A) and genus level (C). Principal Coordinates Analysis (PCoA) based on Bray-Curtis distances illustrates the distinct microbial architecture among the six gut segments at the phylum level (B) and genus level (D). The ten most abundant bacterial taxa at the phylum level (E) and genus level (F) were compared across gut segments using the Kruskal-Wallis H test, revealing significant differences in community composition (*p* < 0.05).

### 2.3. Hematology analysis

Fresh jugular blood was collected into EDTA-anticoagulant tubes (KONSFI, China) for complete blood counts (CBC) analysis using the ProCyte Dx hematology analyzer (IDEXX Laboratories, USA) within 4 h. A second aliquot was drawn into lithium heparin-anticoagulant tubes (KONSFI, China), and plasma was separated by centrifugation at 3,000 × *g* for 10 min at room temperature for subsequent biochemical analysis using the Catalyst One (IDEXX Laboratories, USA) analyzer within 4 h.

### 2.4. Serum vitamins and minerals analysis

Serum prepared from non-anticoagulant blood samples was collected after clotting at room temperature for 30 min, followed by centrifugation (3,000 × *g*, 10 min), and then analyzed for concentrations of vitamins (A, B1, B2, B6, B9, B12, C, D3, E) and minerals (Fe, Mg, Ca, Zn, Cu, Mn) using an automatic vitamins & trace elements analyzer (WJ-9600, Anhui Jiulu Biotechnology, China), following the manufacturer’s instructions. All assays included the manufacturer-provided internal quality controls.

### 2.5. DNA extraction, PCR amplification, and 16S rRNA sequencing

Approximately 1 gm of homogenized gastrointestinal digesta was subjected to bead-beating extraction with SDS/GITC and phenol-chloroform isoamyl alcohol. DNA was precipitated with isopropanol/sodium acetate precipitation, washed with 70% ethanol, and resuspended in TE buffer (pH 8.0). DNA concentration and purity (A260/A280 ratio of 1.8–2.0) were assessed using a NanoDrop 2000 spectrophotometer (Thermo Fisher Scientific, USA), and the DNA was stored at –20°C until use. The V3-V4 hypervariable region of the bacterial *16S rRNA* gene, yielding a 468 bp amplicon. PCR was performed in triplicate with high-fidelity DNA polymerase (Takara Bio, China) and amplified using primers 338F/806R (338F: 5’-ACT CCT ACG GGA GGC AGC AG-3’ and 806R: 5’-GGA CTA CHV GGG TWT CTA AT-3’), and amplicons were pooled and purified using the DNA Gel Extraction Kit (Axygen, USA). Libraries were quantified, normalized, and sequenced on the Illumina MiSeq platform (2 × 300 bp paired-end) to a target depth of ≥ 50,000 reads per sample. All samples met this threshold after quality filtering. Raw sequences were processed using QIIME 2 (v2022.2). After demultiplexing, reads were denoised and merged into amplicon sequence variants (ASVs) using the DADA2 plugin. Chimeric sequences were removed during this step. Taxonomy was assigned to ASVs against the SILVA 138 reference database (99% similarity). Only ASVs present in at least two samples or with a relative abundance > 0.01% across the dataset were retained for downstream analysis.

### 2.6. Data processing and statistical analysis

Body morphometric measurements and hematological parameters were summarized using descriptive statistics. Results are presented as mean ± standard deviation (SD).

Paired-end reads were merged using FLASH v1.2.7. Quality filtering was performed with FastPv0.19. 6. High-quality reads were clustered into OTUs (97% similarity) using UPARSE v7.1, with taxonomy assigned using RDP Classifier v2.2 against SILVA v138 (confidence ≥ 0.7). Data were rarefied to 20,000 reads/sample, ensuring ≥ 99.9% Good coverage. Alpha diversity metrics (observed OTUs, Chao1, and Shannon) and rarefaction curves were computed with Mothur v1.30. 1. Beta diversity was assessed via Bray–Curtis PCoA using the vegan R package, with group differences tested by PERMANOVA (999 permutations). Stacked bar chart showing the relative abundance (%) of bacterial taxa at the phylum and genus level; only mean relative abundances ≥ 1% across all samples are displayed, with others grouped as “others”. Differential abundance analyses from phylum to genus were conducted using LEfSe (LDA > 2, *p* < 0.05). Functional potential was predicted with PICRUSt2.

Association between key bacterial genera and host traits, including body weight, linear morphological indices, hematological parameters, and serum nutrient concentrations were calculated using Spearman’s rank correlations. Significant associations were defined as |*r*| > 0.8 with Benjamini-Hochberg-adjusted *p* < 0.05. Microbial correlation networks were visualized with igraph and ggraph in R v4.5.0 using a Fruchterman-Reingold layout.

## 3. Results

### 3.1. Body weight and body conformation

The average body weight (BW) of the castrated adult crossbred Tibetan goats was 58.42 kg, with minimal variation observed across the recorded morphological traits (Table 1). Breed-related body measurements, including withers height (WH; mean 76.04 cm), heart girth (HG; mean 30.66 cm), and body diagonal length (BDL; mean 84.74 cm), defined the overall body morphology of this population at 3.5 years old. Morphometric ratios relative to body weight were consistent across individuals, and the mean body condition score was 3.5 (on a 5-point scale), indicating generally stable health and adequate physical condition.

**Table 1.** Body conformation, haemato-biochemical, and serum nutrient profile of adult Tibetan goats (castrated) under investigation (*n* = 5).

Body conformation	Mean ± SD	CBC	Mean ± SD (Ref)	Biochemistry	Mean ± SD (Ref)	Nutrients	Mean ± SD (Ref)
Body weight (BW) (Kg)	58.42 ± 2.17	RBC (10^12^/L)	21.5 ± 0.28(10.32–24.43)	TP (gm/l)	76.67± 3.21(64–78)	Vitamin A (μg/l)	1175.93 ± 319.7(830–3070)
Withers height (WH) (cm)	76.04 ± 1.53	HGB (gm/dl)	11.6 ± 1.04(8.9–13.8)	ALB (gm/l)	30.67 ± 3.51(28–38)	Vitamin B1(μg/l)	66.57 ± 12.03(46–112)
Rump height (RH) (cm)	72.96 ± 1.15	HCT (%)	28.93 ± 5.36(22–39)	GLO (gm/l)	45.67 ± 5.03(9.9–50)	Vitamin B2 (ng/ml)	366.93 ± 13.15(185–420)
Heart girth (HG) (cm)	30.66 ± 2.97	MCV (fl)	13.47 ± 2.5(14–22.3)	A/G	0.5 ± 0.21(0.76–1.85)	Vitamin B6 (ng/ml)	116.63 ± 58.78(40–270)
Abdomen depth (AD) (cm)	44.04 ± 3.93	MCH (pg)	5.37 ± 0.47(5.0–7.0)	ALT (U/l)	28.67 ± 7.37(23–44)	Vitamin B9 (ng/ml)	7.53 ± 0.55(3–10)
Chest width (CW) (cm)	31.44 ± 1.68	MCHC (gm/dl)	40.6 ± 4.59(32–34)	CREA (mmol/l)	63.33 ± 5.51(53–124)	Vitamin B12 (pg/ml)	729.2 ± 32.9(450–800)
Chest circumference (CC) (cm)	83.08 ± 1.01	RETIC (K/μl)	7.9 ± 2.51(0–15)	CHOL (mg/dl)	1.91 ± 0.36(1.63–2.79)	Vitamin C (ng/ml)	4897.9 ± 638.4(2992–9891)
Abdomen circumference (AC) (cm)	106.38 ± 1.59	WBC(10^9^/L)	14.87 ± 0.85(6.03–19.58)	TRIG (mmol/l)	0.15 ± 0.05(0.11–0.33)	Vitamin D3 (nmol/l)	172.6 ± 120.17(48–350)
Body diagonal length (BDL) (cm)	84.74 ± 2.71	NEU(10^9^/L)	5.99 ± 1.47(1.72–10.61)	GLU (mmol/l)	8.06 ± 3.01(3.00–5.17)	Vitamin E (mg/l)	12.3 ± 7.4(3–24)
Rump length (RL) (cm)	18.34 ± 1.09	LYM(10^9^/L)	6.58 ± 1.43(2.68–11.54)			Vitamin K1 (μg/l)	1.17 ± 0.4(0.8–2)
Hook bone width (HBW) (cm)	17.58 ± 1.27	MONO(10^9^/L)	1.12 ± 0.34(0.06–0.89)			Fe (μmol/l)	30.84 ± 10(15–45)
Pin bone width (PBW) (cm)	26.78 ± 1.41	EOS(10^9^/L)	1.08 ± 0.14(0.03–1.29)			Magnesium (mmol/l)	1.04 ± 0.42(0.6–1.3)
Rump width (RW) (cm)	16.08 ± 1.17	BASO(10^9^/L)	0.09 ± 0.09(0.00–0.24)			Calcium (mmol/l)	2.45 ± 0.44(2.4–3.0)
		PLT (K/μl)	454.33 ± 97.57(246–912)			Zinc (μmol/l)	10.61 ± 2.95(7.7–19.9)
		MPV (fl)	7.77 ± 0.06(N/A)			Copper (μmol/l)	15.73 ± 0.88(15.7–18.9)
						Manganese (ng/ml)	7.5 ± 5(0–20)

### 3.2. Hematological and serum nutrient profile

The majority of CBC parameters and biochemical parameters in crossbred Tibetan goats were within established reference ranges (Table 1), except for mean corpuscular volume (MCV), mean corpuscular hemoglobin concentration (MCHC), and monocyte count (MONO). Red blood cell count (RBC), hemoglobin concentration (HGB), hematocrit (HCT), and reticulocyte count (RETIC) were all within normal limits. However, a reduced MCV (13.47 ± 2.5 fl) accompanied by an elevated MCHC (40.6 ± 4.59 gm/dl) may be suggestive of mild spherocytosis. Additionally, monocyte count was moderately increased (1.12 ± 0.34 10^9^/L), while white blood cell (WBC), neutrophil (NEU), lymphocyte (LYM), eosinophil (EOS), and basophil (BASO) levels remained within normal ranges. This elevation in monocytes may reflect physiological stress or a recent subclinical inflammatory response. Regarding serum biochemistry, blood glucose concentrations were elevated above typical reference levels, potentially due to acute stress prior to slaughter. Other biochemical markers, including total protein (TP), albumin (ALB), alanine transaminase (ALT), total cholesterol (CHOL), triglycerides (TRIG), and creatinine (CREA), were within physiological ranges for other goat populations.

Although most vitamin concentrations were within the reference ranges reported for ruminants [12], notable inter-individual variation was observed in vitamin B6 (116.63 ± 58.78 ng/ml), vitamin D3 (172.6 ± 120.17 nmol/l), and vitamin E (12.3 ± 7.4 mg/l), indicating possible differences in dietary absorption efficiency (Table 1). Calcium (2.45 ± 0.44 mmol/l), copper (15.73 ± 0.88 μmol/l), zinc (10.61 ± 2.95 μmol/l), and manganese (7.5 ± 5.0 ng/ml) levels in several individuals were near the lower limits of standard ranges (Table 1), suggesting a potential mineral imbalance. In contrast, iron and vitamin B12 concentrations remained within the mid-to-upper normal range, indicating sufficient nutrient intake and a low likelihood of erythropoietic impairment.

### 3.3. Taxonomical diversity and comparative compositions of gut microbiota

**Supplementary Figure 2.**
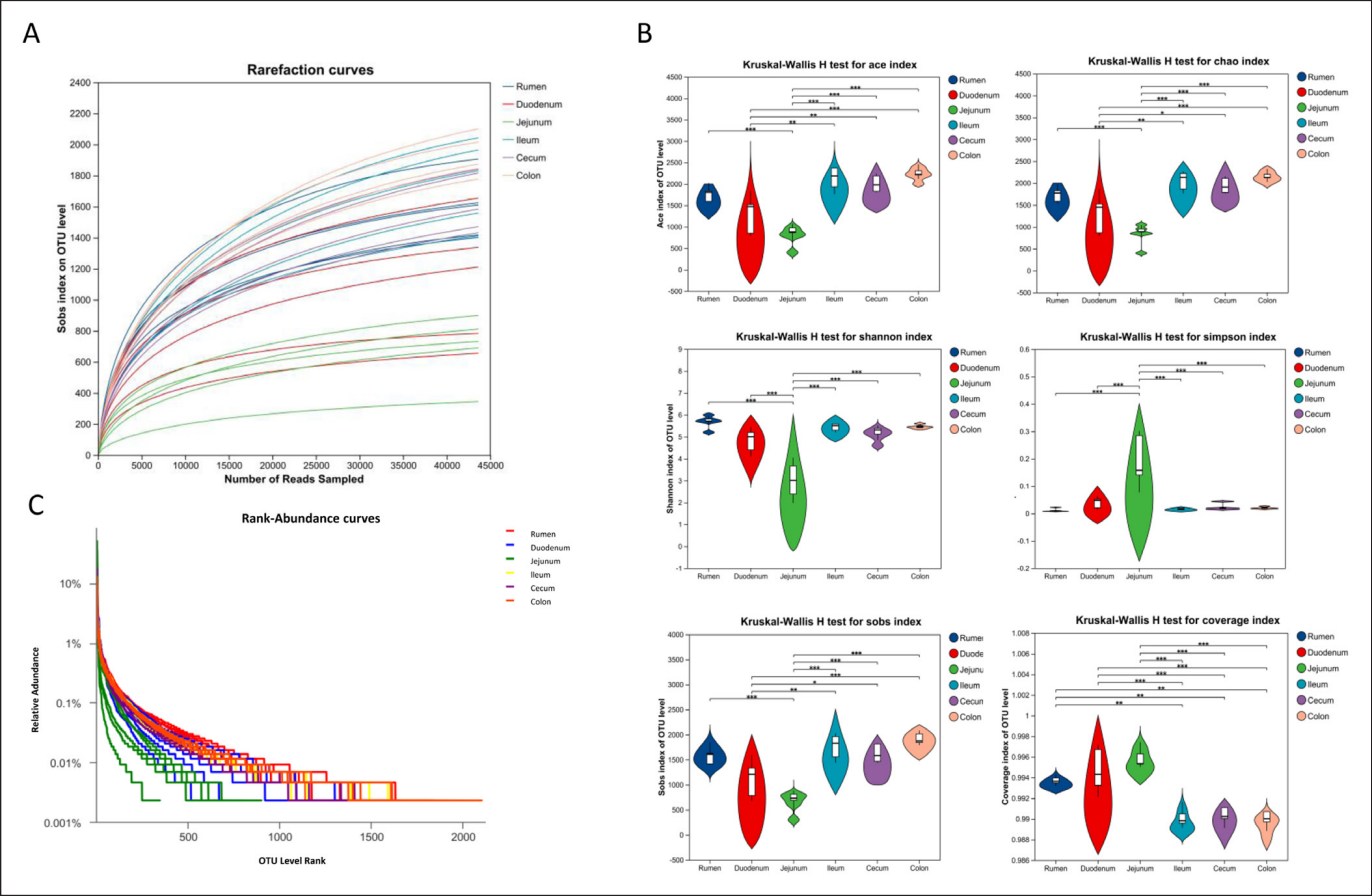
Charts illustrating sequencing depth, species richness and evenness, and alpha diversity across gastrointestinal segments. Rarefaction curves for samples from six gastrointestinal regions, demonstrating sequencing depth and species richness (A). Comparison of alpha diversity indices among the six gut segments, analyzed using the Kruskal–Wallis H test (B). Rank-abundance curves depicting species richness and evenness in microbial communities from each gastrointestinal region (C).

In this study, 30 digesta samples were collected from the rumen, duodenum, jejunum, ileum, cecum, and colon of crossbred Tibetan goats. Following quality control, a total of 2,018,770 high-quality sequences were retained, with an average of 67,292 ± 10,691 reads per sample. Non-redundant sequences were clustered into operational taxonomic units (OTUs) at a 97% similarity threshold, yielding 43,665 OTUs. Rarefaction curves were generated to assess sequencing depth across gastrointestinal regions (Supplementary Figures 2A, 2C), with coverage values exceeding 0.99 for all samples, indicating that the sequencing effort was sufficient to capture the majority of bacterial diversity. In total, 256 bacterial genera representing 16 phyla were identified across all gut segments. Alpha diversity indices, including ACE, Shannon, Simpson, Chao1, and Sobs, varied significantly among gastrointestinal (GI) tract segments (*p* < 0.001), indicating notable differences in microbial richness and diversity. The rumen, ileum, cecum, and colon exhibited higher richness and community complexity, as reflected by elevated ACE and Chao1 values, whereas the duodenum and jejunum showed lower diversity.

Across all sampled regions, Firmicutes (mean abundance, 61.3%) and Bacteroidota (20.8%) were the dominant phyla. Firmicutes were especially enriched in the ileum (68.3%), cecum (66.7%), and colon (72.7%). Actinobacteriota were more abundant in the duodenum (19.8%) and jejunum (26.8%), while Proteobacteria were also prominent in the jejunum (average 19.8%) and showed high inter-individual variation (Figure 1A). The Firmicutes/Bacteroidota (F/B) ratio increased progressively from the rumen to the hindgut, with the highest ratio observed in the small intestine due to a pronounced reduction in Bacteroidota. At the genus level, the *Christensenellaceae_R-7_group*, Prevotella, Monoglobus, Ruminococcus, the NK4A214 group, and UCG-005 were prevalent in the ileum, cecum, and colon. Bacteroides and Akkermansia were also more abundant in the hindgut, while *Aeriscardovia* and *Streptococcus* were enriched in the foregut. The rumen contained distinct genera such as Prevotellaceae UCG-003 and *Butyrivibrio*, which were less represented or absent in other segments. The jejunum exhibited the lowest and most variable microbial diversity among all regions (Figure 1C). Principal coordinates analysis (PCoA) of beta diversity demonstrated clear differentiation in microbial community composition across GI segments at both the phylum and genus levels (PERMANOVA, *p* < 0.01) (Figures 1B, D), reflecting the region-specific structure of the gut microbiota.

Based on these results, differences in microbial composition were further examined using the Kruskal-Wallis H test (Figures 1E, F). At the phylum level, the relative abundances of Bacteroidota, Firmicutes, Actinobacteriota, Desulfobacterota, and Verrucomicrobiota differed significantly among gastrointestinal regions (*p* < 0.05). At the genus level, significant variation was also observed in the abundances of core genera, including *Christensenellaceae_R-7_group*, Prevotella, NK4A214_group, Akkermansia, and Romboutsia (*p* < 0.05).

### 3.4. Interaction networks between microbiota and host traits along the gut segments

An exploratory correlation analysis of microbial genera and host physiological traits revealed region-specific patterns. Dominant genera, including Akkermansia, Aeriscardovia, Bacteroides, *Christensenellaceae_R-7_group*, Eubacterium coprostanoligenes group, Monoglobus, NK4A214_group, Prevotella, Rikenellaceae_RC9_group, *Romboutsia, Ruminococcus, Streptococcus*, and UCG-005, were significantly associated with parameters related to growth, metabolism, hematological status, immune response, and micronutrient levels.

**Figure 2.**
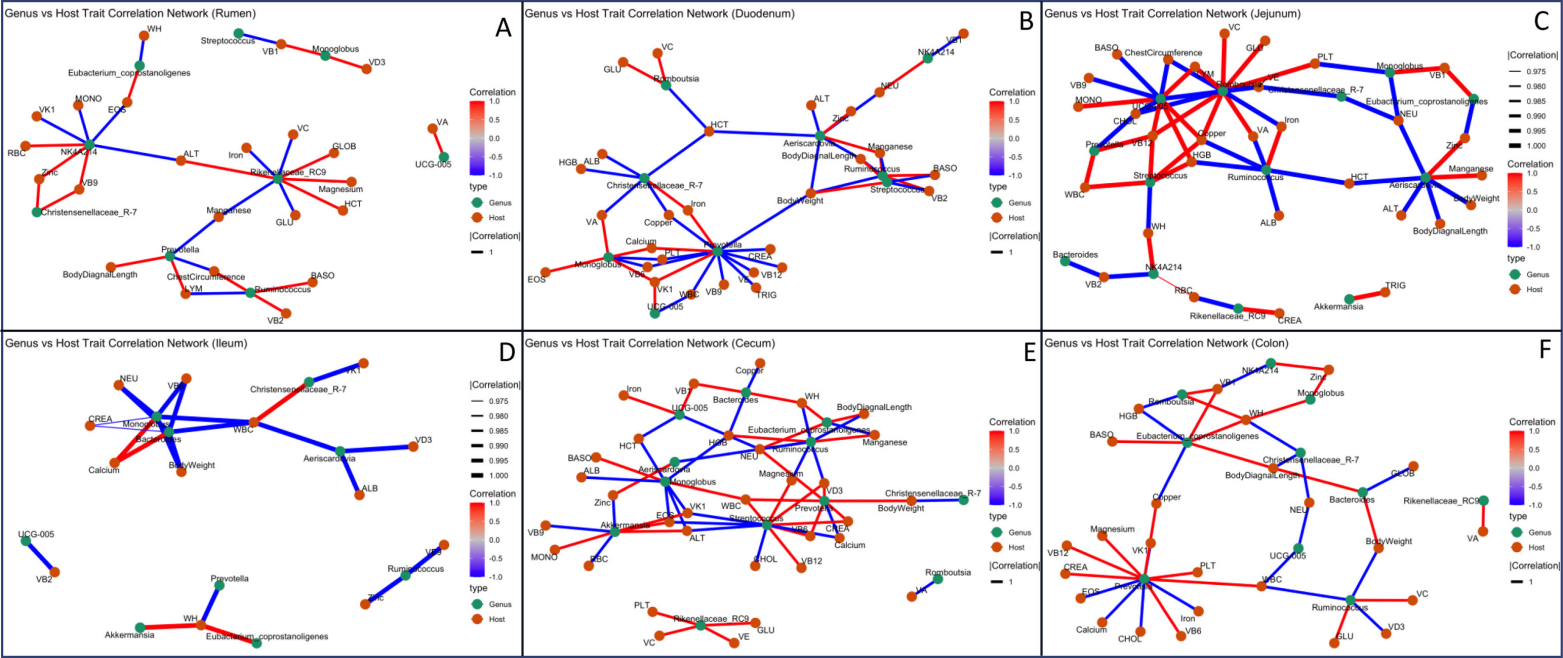
**Networks of gut microbiota-host trait interactions across gastrointestinal segments.** Panels A–F represent the rumen, duodenum, jejunum, ileum, cecum, and colon, respectively. Nodes represent bacterial genera (orange) or host traits (green); node size reflects mean relative abundance (microbes) or standardized trait value (host). Edges indicate significant Spearman correlations (Benjamini–Hochberg–corrected *p* < 0.05); red = positive, blue = negative; edge thickness reflects correlation strength (|*ρ*| ≥ 0.7 shown).

In the rumen, the NK4A214_group, Rikenellaceae_RC9_group, and Prevotella showed correlations with body diagonal length (BDL), blood glucose (GLU), alanine transaminase (ALT), red blood cell count (RBC), hematocrit (HCT), lymphocyte (LYM), and levels of iron (Fe), magnesium (Mg), zinc (Zn), vitamin C (VC), and vitamin K1 (VK1) (Figure 2A). In the duodenum and jejunum, genera such as Aeriscardovia, *Christensenellaceae_R-7_group, Streptococcus*, Monoglobus, Prevotella, *Ruminococcus*, and UCG-005 were strongly associated with body weight (BW), BDL, white blood cell count (WBC), platelet (PLT), ALT, creatinine (CREA), triglycerides (TRIG), and vitamins A (VA), B6 (VB6), and B12 (VB12), along with minerals such as calcium (Ca), copper (Cu), iron (Fe), and manganese (Mn) (Figures 2B, C). In the ileum, fewer microbial-host interactions were identified. Aeriscardovia, Monoglobus, and Bacteroides were among the primary genera associated with limited host traits (Figure 2D). In contrast, the cecum and colon exhibited more extensive associations. Genera including Bacteroides, Akkermansia, Eubacterium coprostanoligenes group, Monoglobus, Prevotella, Ruminococcus, and Streptococcus were significantly correlated with traits such as withers height (WH), hemoglobin concentration (HGB), WBC, neutrophil count (NEU), ALT, and levels of vitamins B1 (VB1), D3 (VD3), and K1 (VK1) (Figures 2E, F).

### 3.5. Seasonal variation in fecal microbiota composition and KEGG pathway predictions

**Figure 3.**
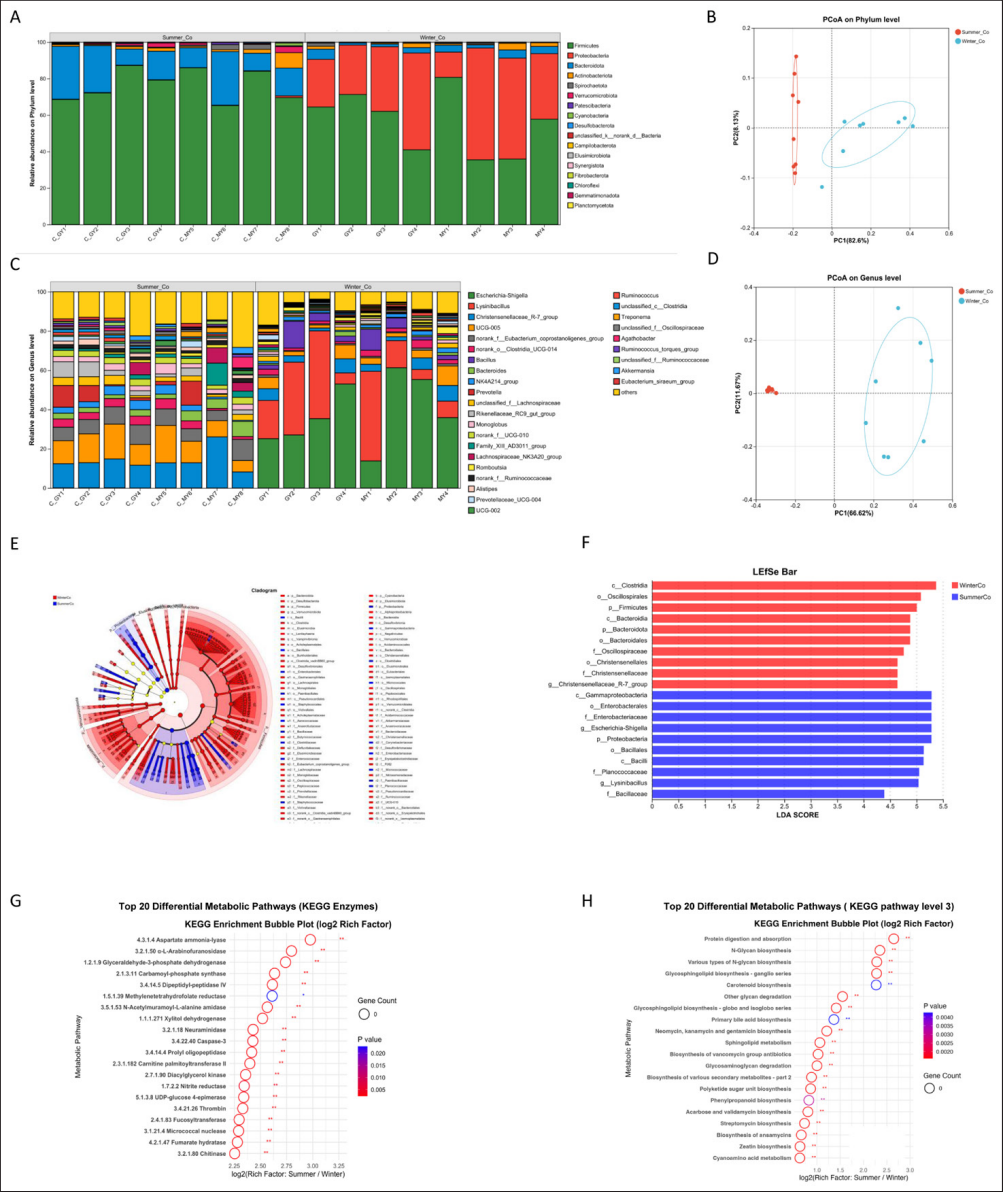
Seasonal variation in fecal microbiota composition and predicted functional potential between winter and summer in Tibetan goats. Stacked bar chart showing microbial composition at the phylum level (A) and at the genus level (B). Principal Coordinates Analysis (PCoA) based on Bray-Curtis distances illustrating seasonal differences at the phylum level (C) and at the genus level (D) between winter and summer. Differences among season-enriched taxa and genera are visualized by the LEfSe Cladogram (E) and LEfSe Bar Plot (F), showing significant seasonal variation between winter and summer (LDA SCORE > 4). Top 20 KEGG enzyme functions (G) and top 20 KEGG metabolic pathways (H) differed in relative abundance with significant seasonal shifts (summer/winter). Bar plots and bubble charts indicate relative abundance and statistical significance (*p* < 0.01).

At the phylum level, Firmicutes (35.5–80.7%) and Proteobacteria (26.1–61.3%) dominated the fecal microbiota in winter, whereas Firmicutes (68.5–87.2%) and Bacteroidota (8.8–29.0%) were more prevalent in summer (Figure 3A). A pronounced seasonal shift was observed in the Firmicutes/Bacteroidota (F/B) ratio, which ranged from 8.02 to 64.73 in winter but was markedly lower (2.37 to 8.74) in summer. At the genus level, fecal microbiota in winter was enriched in *Escherichia-Shigella* (13.8–61.2%), *Lysinibacillus* (5.2–45.7%), *Christensenellaceae_R-7_group* (3.3–8.0%), *UCG-005* (2.1–9.9%), and *Bacillus* (2.2–13.7%), many of which include opportunistic or stress-tolerant taxa. In contrast, summer microbiota showed higher relative abundances of *Christensenellaceae_R-7_group* (8.2–26.0%), *UCG-005* (5.8–19.1%), *Eubacterium_coprostanoligenes_group* (5.3–7.8%), and *Prevotella* (1.3–12.3%) (Figure 3C). Notably, Principal Coordinates Analysis (PCoA) based on Bray-Curtis dissimilarity confirmed significant seasonal separation of microbial communities at both phylum and genus levels (PERMANOVA, *p* < 0.01) (Figures 3B, D), underscoring that seasonal factors exert a strong influence on gut microbial ecology.

LEfSe analysis identified distinct seasonal taxa biomarkers (Figure 3E). Winter-enriched taxa were primarily from Proteobacteria (e.g., *Escherichia-Shigella*), Actinobacteriota, and spore-forming Firmicutes (e.g., *Lysinibacillus* and *Bacillus*). In contrast, summer-enriched taxa included *Prevotella, Christensenellaceae_R-7_group*, and *members of Lachnospiraceae* genera. The high LDA scores for these taxa further support their role as seasonal indicators (Figure 3F).

Predicted KEGG pathway via PICRUSt2 revealed significant seasonal differences in microbial functional potential (Figures 3G, 3H). Enzymes involved in energy metabolism (e.g., fumarate hydratase, xylitol dehydrogenase) and stress-related processes (e.g., caspase-3, linked to apoptosis regulation) were differentially abundant (*p* < 0.01). Winter samples showed enrichment in pathways for streptomycin biosynthesis, glycan degradation, and secondary metabolite production, possibly reflecting microbial defense mechanisms or adaptation to low-quality forage. Conversely, summer microbiota exhibited higher predicted activity in N-glycan biosynthesis and primary bile acid metabolism, pathways that may enhance nutrient absorption and modulate host lipid metabolism during periods of abundant forage intake.

## 4. Discussion

This study provides the first integrated clinical and microbiome characterization of crossbred Tibetan goats managed under high-altitude, year-round free-range grazing systems. The animals maintained acceptable body condition scores and largely stable hematological parameters despite exposure to hypobaric hypoxia, temperature fluctuations, and seasonal forage limitations, which may reflect physiological adaptation and productive potential in extensive management settings [6, 13]. Their morphometric traits and body weights were comparable to those of intensively reared meat breeds such as Nanjiang Yellow and Jianzhou Big-Ear goats [14, 15], suggesting that this crossbreeding strategy may preserve growth potential (as in Boer goats) while retaining adaptation to extensive conditions at high altitudes [16]. Similar resilience has been reported in Tibetan sheep and yaks [17, 18], but it is rarely documented in cross-bred Tibetan goats. Notably, subtle nutrient deviations in blood profiles likely reflect seasonal declines in forage mineral content rather than underlying disease, consistent with the observed geophagy. While these fluctuations did not impair short-term performance, chronic deficiencies could eventually affect growth, reproductive efficiency, and immune competence [19]. This underscores the need for context-specific nutritional support, particularly during cold periods when native forage is insufficient.

Gut microbiota profiling across six gastrointestinal segments revealed marked spatial heterogeneity, with the highest alpha diversity in the hindgut and a progressive increase in the Firmicutes/Bacteroidota (F/B) from foregut to hindgut. This gradient aligns with the high-fiber, lignocellulosic nature of the alpine shrub-grassland diet and mirrors patterns observed in yaks and Tibetan sheep [18, 20], suggesting convergent microbial strategies among high-altitude ruminants. The elevated F/B ratio, particularly in the colon, may enhance fermentative capacity and energy harvest from recalcitrant plant material, a critical adaptation under nutrient-limited conditions [21, 22, 23]. However, it should be noted that the F/B ratio should be interpreted alongside taxon-specific roles and host physiology when it is implicated in the metabolic function [24, 25]. The jejunum harbored the most distinct microbial community, enriched in genera such as Aeriscardovia and Streptococcus, which correlated with circulating B-vitamin levels and metabolic markers [22, 26]. Given the jejunum’s primary role in nutrient absorption and mucosal immunity [23], this finding supports the hypothesis that local microbial assemblages may modulate host nutrient sensing and barrier function.

Microbiota-host interaction networks revealed strong segment-specific associations, underscoring the gut microbiota’s influence on physiological processes. In the rumen, taxa including the *NK4A214_group* and *Rikenellaceae_RC9_group* were positively associated with morphometric indices and serum concentrations of iron, magnesium, zinc, and vitamins, suggesting a potential role in mineral mobilization or bioavailability [23, 27]. While these correlations do not establish causality, they are consistent with emerging evidence from yak studies linking rumen microbes to systemic micronutrient status [21]. In the small intestine, especially the jejunum, Aeriscardovia, *Streptococcus*, and UCG-005 were associated with vitamin B levels and metabolic indicators, highlighting their roles in nutrient sensing and mucosal immunity [26]. The hindgut microbiota showed strong associations with fat-soluble vitamins and immune markers, reflecting its fermentative and barrier-supporting functions [28, 29]. These associations also suggested that gut microbiota contribute to host physiological homeostasis via both metabolic and immunological mechanisms. A limitation of this study is the small sample size, as data were derived from only five goats. Nevertheless, the findings offer preliminary insights into the caprine gut microbiota atlas and its association with key physiological parameters. Larger, controlled studies are needed to confirm these observations and evaluate their generalizability across diverse goat populations.

Seasonal shifts in rectal fecal microbiota were pronounced. Winter communities were enriched in *Clostridia, Oscillospirales*, and *Christensenellaceae_R-7_group*, implicated in fiber fermentation and energy conservation during nutritional stress [30]. Concurrently, functional predictions indicated upregulation of pathways for protein fermentation and bile acid metabolism, possibly compensating for reduced dietary energy intake. In contrast, summer microbiota was characterized by decreased abundances of *Escherichia-Shigella* and *Lysinibacillus*, likely reflecting shifts due to higher carbohydrate intake and possible enrichment of opportunistic bacteria [31]. A higher F/B ratio and relative abundances of SCFA-producing taxa in summer, alongside increased predicted capacity for secondary metabolite biosynthesis (e.g., polyketides, streptomycin derivatives), likely reflect greater intake of diverse, carbohydrate-rich forage. The summer-associated rise in SCFA producers may support gut barrier integrity and anti-inflammatory effects [32, 33], whereas the winter increase in Proteobacteria, including *Escherichia* and *Shigella*, could reflect transient dysbiosis under cold stress, as observed in other alpine herbivores [34, 35]. Importantly, these microbiota restructurings may be the integrated outcome of concurrent climate, environmental, and dietary changes, as well as host status. Future work should employ longitudinal sampling, paired with forage nutrient analysis, metabolomics, and measures of feed efficiency, to clarify whether specific microbial features can serve as biomarkers for stress resilience or husbandry productivity [36].

## 5. Conclusions

In conclusion, this study advances understanding of caprine adaptation to high-altitude grazing by linking spatially resolved gut microbiota profiles with host physiological indicators across seasons and offers new insights into the mechanisms underpinning nutrient utilization in extensive systems. These findings highlight the potential for microbiome-informed management, such as seasonal mineral supplementation or targeted forage enhancement, to support both animal health and ecological sustainability in vulnerable alpine grasslands.

## Data Availability

The data presented in this study are available from the corresponding author upon reasonable request.

## List of abbreviations

ACE, Abundance-based Coverage Estimator; KEGG, Kyoto Encyclopedia of Genes and Genomes; LEfSe, Linear discriminant analysis Effect Size; PCR, polymerase chain reaction; SCFA, short-chain fatty acid.
